# Notices, Reviews, &c.

**Published:** 1859-04

**Authors:** 


### NOTICES, REVIEWS, &c.

We had just finished reading the "proof" of our article—"A Daily Religious Newspaper," when a gentleman informed us that a hundred and fifty thousand dollars were secured for commencing such an enterprise in New York, and that it would shortly be put in execution. Simultaneously in London, a million and a half of dollars have been secured for making a religious daily of "*The Dial.*" Who shall not say "The day breaketh."

The *Westminster Review* for January, reprinted by Leonard Scott & Co., New York, at $3 a year, has an able and extended article on Chloroform and other anaesthetics, which the medical scholar will read with interest and profit.

*House Architecture*, Fowler & Wells, New York, sent pp. for 30 and 50 cents, being No. 1, of Rural Manuals, useful and practical.

*The British & Foreign Medico Chirurgical Review*, published quarterly by the Messrs. Wood, 389 Broadway, New York, $3 a year, postage free, to all advance subscribers, is a standard and sterling publication. The No. for Jan., 1859, is of unusual interest: sanatory science, obstetrics, cerebral physiology, influence of climate on disease, therapeutics of electricity, the antagonism of ague and consumption discussed, &c., &c. The Messrs. W. offer 23 new medical works from London, with 300 vols. in French, at reduced prices; also sixteen different works on diseases of the ear, ninety on the eye, and seventy-six on the teeth, with prices attached. They promptly order medical works from all parts of the world.

*Boston Medical & Surgical Journal*, $3 a year, for March, is enriched by observations of M. Trosseau, on asthma; himself a sufferer from that disease.

A line or two from the eastern shore of Maryland, telling how much interested two deaf and dumb mutes were in reading one or two articles in a stray *Journal of Health*, encourages us to hope that we are not wholly living to or for ourselves.

The unknown (to us) Editor of the *United States Hotel Directory*, weekly, $2 a year, New York, has our thanks and cordial approbation for the real excellence of the reading matter of his paper, original and selected; we hope he will continue the same.

*Medical and Surgical Journal*, weekly, Philadelphia; increases in interest with the evident thrift of the new enterprize of Drs. Butler and Lewis, judging from its mechanical improvement.

The *American Agricultural Monthly*, $1 a year. Orange Judd, New York, is a marvel of the age.

